# IL-17 Mildly Rescued the Impaired Proliferation of Alveolar Epithelial Cells Induced by LCN2 Overexpression

**DOI:** 10.1155/2024/9284430

**Published:** 2024-08-06

**Authors:** Tingting Lv, Ziliang Hou, Kaiyuan Yang, Jinxiang Wang

**Affiliations:** Department of Respiratory and Critical Care Medicine Beijing Luhe Hospital Capital Medical University, Beijing 101100, China

## Abstract

**Introduction:**

The impaired proliferative capacity of alveolar epithelial cells after injury is an important factor causing epithelial repair dysfunction, leading to the occurrence of idiopathic pulmonary fibrosis (IPF). Alveolar type 2 (AT2) cells as the stem cells of alveolar epithelium participate in the repair process after alveolar injury. Lipocalin-2 (LCN2) participates in multiple processes regulating the pathological process of alveolar epithelial cells, but the mechanisms involved are still unclear.

**Method:**

We used a BLM-treated mouse model to characterize the expression of LCN2 in lung fibrosis regions and analyzed the location of LCN2 in alveolar epithelial cells. Moreover, human pulmonary alveolar epithelial cells (HPAEpiCs) were transfected with the LCN2 overexpression plasmid vector in vitro. Recombinant human interleukin-17 (IL-17) protein (rhIL-17) at different concentrations was administered to intervene in HPAEpiCs, observing cell viability and analyzing the concentration-dependent effect of IL-17.

**Results:**

LCN2 was increased in the alveolar epithelium post-BLM injury, and highly expressed LCN2 was mainly concentrated on AT2 cells in BLM-injured lungs. Meanwhile, LCN2-overexpressing HPAEpiCs showed impaired cell viability and cell growth. HPAEpiC intervention with rhIL-17 mildly rescued the impaired cell proliferation induced by LCN2 overexpression, and the effect of IL-17 intervention was partially concentration-dependent.

**Conclusions:**

The results revealed the reversed effect of IL-17 on the impaired proliferative capacity of the alveolar epithelium induced by LCN2 overexpression. The target alveolar epithelial cells regulated by this process were AT2 cells, providing new clues for alveolar epithelium repair after injury and the treatment of lung injury diseases.

## 1. Introduction

The pulmonary alveolar epithelium consists of alveolar type 1 (AT1) and type 2 (AT2) cells, which function in the blood-gas exchange process and are a crucial barrier protecting against the invasion of harmful factors [1]. Chronic damage to the alveolar epithelium has been demonstrated to be involved in the development of several lung diseases involved in the pathogenesis of IPF [2]. The mechanisms contributing to the alveolar epithelium damage in IPF are complex, including abnormal cellular regulation and metabolic processes, such as impaired cellular regenerative capacity, increased apoptosis, and accelerated senescence [2, 3].

Later studies utilizing lineage-tracing experiments in transgenic Sftpc-CreER; Rosa-Tm reporter mice confirmed that AT2 cells served as the stem/progenitor cells of the alveolar epithelium, and lung injury can trigger AT2 cells to self-renew or transdifferentiate into AT1 cells, thereby maintaining the alveolar epithelial function upon both homeostasis and injury state [4, 5]. Experimental results based on BLM-induced alveolar epithelial injury in mice confirmed that the proportion of AT2 cells in the alveolar epithelium decreased significantly after lung injury [4]. In addition, IPF, resulting from repeated injury and loss of repair capacity, is a commonly observed dysregulated function of the alveolar epithelium where the normal function of the alveolar epithelium for gas exchange cannot be maintained [2]. Therefore, to some extent, impaired regenerative capacity of the alveolar epithelium may contribute to the development of IPF.

According to the differential gene expression of our previous single-cell RNA sequencing analysis of AT2 cells after BLM injury, we concluded that genes participating in immune responses were highly expressed in alveolar epithelial cells postinjury, such as LCN2 [3]. Lipocalin-2 (LCN2) is a biologically active protein that binds to specific receptors in neutrophils, macrophages, and epithelial cells, showing antimicrobial effects and activating inflammatory factors [6]. Relevant studies found that LCN2 was significantly upregulated in mouse lung epithelium after exposure to inhalation of ultrafine carbon particles and in human lung tissue with severe inflammation, but some alveolar cells in uninflamed lungs showed weak staining of LCN2 [7, 8]. Thus, we concluded that LCN2 may participate in regulating the pathological or inflammatory processes of alveolar epithelial cells, but the probable mechanism affecting that process has not been investigated.

Current studies have found that LCN2 is a typical target gene of IL-17, which is one of more than 30 types of interleukin discovered and secreted by CD4^+^ T cells [9]. Studies on the relationship between IL-17A and LCN2 have focused on chronic inflammatory diseases, including enteritis, dermatitis, and lung inflammation [10–12]. IL-17A reportedly promotes increased expression of LCN2 in human neutrophils and remodeling of airway epithelium in patients with chronic obstructive pulmonary disease (COPD) [10]. However, the effect of IL-17 on the expression of LCN2 in alveolar epithelial cells remains unclear.

Here, we first used a BLM-treated mouse model to characterize the expression of LCN2 in the lung fibrosis region and analyzed the location of LCN2 in alveolar epithelial cells. We demonstrated that the expression of LCN2 in AT2 cells was influenced by lung tissue injury. To observe the effect of overexpressed LCN2 on alveolar epithelial cells, LCN2 HPAEpiC plasmids were utilized in vitro to further evaluate cell proliferation. Given the close relationship between LCN2 and IL-17, we then used HPAEpiC intervention with rhIL-17 protein and observed its effect on decreased cell proliferation induced by overexpressed LCN2. Meanwhile, surfactant protein-C (SP-C) served as the surface marker of AT2 cells [4], so Prospc-positive HPAEpiCs demonstrated that the target cells were affected, indicating a revealable directivity to type 2 alveolar epithelium cells in vivo. Thus, our study opens the door for further exploration of the effect of LCN2 on lung injury diseases, such as IPF, and the impact of IL-17 on that process to provide fundamental ideas for the diagnosis and treatment of IPF.

## 2. Materials and Methods

### 2.1. Mice

C57BL/6 male mice were selected for our animal experiments and were purchased from SiPeiFu, Beijing, China. All mouse experiments were performed in accordance with the guidelines for the use and care of laboratory animals. The experimental procedures used in animal studies were approved by Capital Medical University, Beijing. Approval was granted by the Ethics Committee of Capital Medical University (no. AEEI-2023-156). Mice were housed under standard environmental conditions (20–22°C, 12 h/12 h light/dark cycle) and provided food and water ad libitum. Mice were anesthetized using pentobarbital sodium before sacrifice. All mice were acclimated to the laboratory environment for at least 1 week before receiving BLM/vehicle intratracheal instillation.

### 2.2. BLM-Induced Lung Injury and Lung Fibrosis Analysis

The age of the mice in our study was 8–10 weeks. Ten mice in each group (pretreatment body weight was assessed) received (intratracheally) a single dose of BLM (15 mg, Hai Zheng Pfizer Inc, China) 1.5 U/1,000 g body weight. BLM stock solution was dissolved in 5 mg mL^−1^ sterile saline, and aliquots were frozen at −20°C until use. The BLM working solution was prepared immediately before treatment. After intraperitoneal injection with 0.8% sodium pentobarbital (0.8 mL/10 g body weight), mice were intratracheally injected with a single dose of bleomycin (1.5 U kg^−1^ body weight) or saline (vehicle). The BLM/vehicle-treated mice were sacrificed at Day 0 and Day 28 posttreatment (*n* = 10/group). Lung fibrosis was analyzed using a survival curve, H&E staining, and COL1A1 staining. Immunofluorescence staining of COL1A1 was utilized to assess collagen production in lung tissue post-BLM.

### 2.3. Immunohistochemical and Immunofluorescent Staining

The immunoreactivity of LCN2 was detected in paraffin-embedded mouse lung sections using a rabbit anti-mouse LCN2 antibody (1 : 100 dilution, Affinity, USA, DF6816). Positive signals were visualized using HRP-conjugated goat anti-rabbit secondary antibody (1 : 500 dilution). The positive cells were developed by diaminobenzidine (DAB) reagent, and nuclei were stained with hematoxylin. To assess histological changes and to assess the expression of LCN2 (antibody 1 : 100 dilution, Affinity, USA) and Prospc (antibody 1 : 200 dilution, Santa Cruz Biotechnology, Beijing, China, 52–398703) in lung tissue by immunofluorescent staining, images of positive regions were obtained under a Nikon microscope (Nikon, Japan). The counts of total LCN2-positive cells and LCN2 and Prospc double-positive cells were acquired by using ImageJ software.

### 2.4. Cell Culture and Reagents

The HPAEpiC cells were purchased from Otwo Biotech, Shenzhen, China (HTX2419). HPAEpiC cells were grown in DMEM (Gibco, Camarillo, CA, USA, 11995065) supplemented with 10% FBS (Gibco, Camarillo, CA, USA, 164210) and 1% penicillin-streptomycin (10 000 mg mL^−1^ penicillin and 10 mg mL^−1^ streptomycin; Solarbio Life Science, Beijing, China). After trypsinization for 1 minute with 0.05% trypsin-EDTA (Solarbio Life Science, Beijing, China), cells were cultured in a humid incubator (37°C, 5% CO_2_) before use.

### 2.5. Plasmid Transfection In Vitro

HPAEpiCs under transfection were divided into three groups: the control group, the plasmid vector group, and the plasmid LCN2 group. Twenty-four hours before transfection, cells were seeded into 6-well plates. Subsequently, Lipofectamine™ 3000 (Invitrogen, Carlsbad, CA, USA) was diluted with DMEM basal medium and mixed and incubated at room temperature. At the same time, the plasmid vector/plasmid LCN2 was diluted with DMEM basal medium and mixed at room temperature. After 5 minutes, the two mixtures above were mixed at a ratio of 1 : 1, followed by incubation at room temperature for 15 minutes. Next, 500 *μ*L of the mixture was added to each well of a 6-well plate, shaken slightly, and placed in a cell incubator. Six hours later, the DMEM basic medium was replaced with the DMEM complete medium. The cell transfection process was terminated for subsequent experiments.

### 2.6. Immunocytochemistry

Immunocytochemical (ICC) staining was performed on cultured HPAEpiCs grown on sterile coverslips. The coverslips with cells were incubated in rabbit anti-mouse Prospc antibody (antibody 1 : 200 dilution, Santa Cruz Biotechnology, Beijing, China) at 4°C overnight and incubated in goat anti-rabbit secondary antibody (Santa Cruz Biotechnology, Beijing, China) diluted 1 : 200 at 37°C for 2 h. Then, the coverslips were counterstained with 4′,6-diamidino-2-phenylindole (DAPI) (Sigma-Aldrich, USA) for 10 min. The counts of total cells and Prospc-positive cells per 2748 *∗* 2200 pixels were obtained by using ImageJ software.

### 2.7. Cell Viability Assay

CCK8 was applied to assess HPAEpiC proliferation according to the manufacturer's instructions. HPAEpiCs were seeded in a 96-well cell culture plate (4 × 10^4^ cells mL^−1^) and then treated with rhIL-17 (NOVO protein, Beijing, China, C774) at different concentrations (10 ng/ml, 20 ng/ml, and 50 ng/ml). Subsequently, according to the manufacturer's instructions for Cell Counting Kit-8 (CCK8) (Solarbio, Beijing, China, CA1210), 10 *µ*L of CCK-8 was added to the well. After 2 hours, the absorbance was measured using a Genios multifunction reader (BioTek, Vermont, USA) at 450 nm.

### 2.8. EdU Assay

Cell proliferation was measured using an EdU assay kit (MCE, China, HY118411). Cells were seeded into 6-well plates (2 × 10^5^ cells/well) and cultured for 24 hours before the addition of EdU (50 *μ*mol/L). According to the protocols, the cells were then incubated for 2 hours at 37°C, fixed in 4% formaldehyde for 15 minutes, and permeabilized with 0.3% Triton X-100 for 15 minutes at room temperature. After washing with PBS, 430 *µ*L of click reaction buffer, 20 *µ*L of CuSO_4_, 1 *µ*L of azide 488, and 50 *µ*L of click additive solution were added to each well, mixed well, and incubated with EdU for 30 minutes. Subsequently, Hoechst (1 mL/well, 1 : 1000) was added for 10 minutes to visualize the nuclei. Images of cells were obtained under a Nikon microscope (Nikon, Japan). Cell proliferation was analyzed via the EdU-positive cell number ratio for each sample by using ImageJ software.

### 2.9. Statistical Analysis

All experiments were repeated at least three times. Data were analyzed with GraphPad Prism 6.0. Student's *t* test was used to determine significant differences between two groups, and one-way ANOVA with Tukey's post hoc analysis was performed to compare more than two groups. All data are reported as the mean ± SD, and a value of *P*  <  0.05 was considered statistically significant.

## 3. Results

### 3.1. The Expression of LCN2 Was Increased in Mouse Lungs Post-BLM Injury

**Figure 1:**
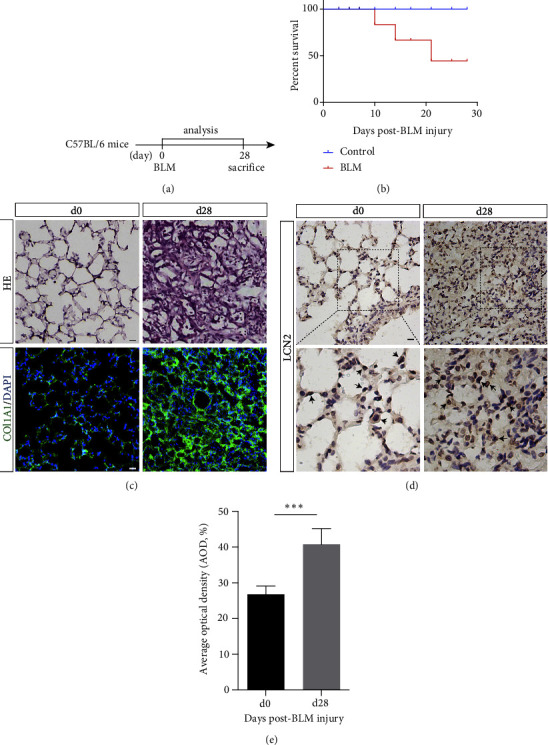
Lcn2 was increased in mice lung post-BLM injury. (a) Timeline of experimental procedure. C57BL/6 mice (6–8 weeks) were subjected to lung injury by intratracheal instillation of BLM (1.5 U kg^−1^), lungs were collected and analyzed at Day 0 and Day 28 post-BLM injury. (b) Mice post-BLM showed lower survival rate as compared to control mice. (c) Mice lungs at Day 0 and Day 28 post-BLM injury were collected for HE staining and immunofluorescent staining of COL1A1. (d, e) Mice lungs at Day 0 and Day 28 post-BLM injury were collected for immunohistochemical staining of LCN2 (d) and average optical density (AOD, %). Data are shown as mean ± SEM, *n* = 10; ^*∗∗∗*^*P* < 0.01. Scale bar, 100 *μ*m.

We utilized the popular lung injury mouse model induced by BLM to verify the close relationship between LCN2 and lung epithelial tissue after injury. The mice were intratracheally instilled with BLM at a dose of 1.5 U kg^−1^ body weight (Figure 1(a)). The death of the BLM group occurred as early as 10 days, and the peak of death was mainly concentrated 10∼20 days after BLM instillation. The survival rate of BLM-treated mice was less than 50% compared with that of vehicle-treated mice (Figure 1(b)).

To evaluate the effectiveness of BLM injury, we performed H&E staining to analyze the histological changes in the lungs. Histological hallmarks can be present, such as the strongly damaged structure of alveoli, alveolar septal edema, and filling with inflammatory cells and collagen fibers (Figure 1(c)). In addition, immunofluorescent staining of collagen type 1 alpha 1 (COL1A1) acted as the marker of collagen. Collagen was used to observe the severity of lung tissue fibrosis, revealing that the levels of COL1A1 in the lungs of mice at post-BLM Day 28 were significantly higher than those at post-BLM Day 0 (Figure 1(c)). To further explore the expression and distribution of LCN2 in the lung alveolar epithelium, we performed immunohistochemistry (IHC) of LCN2. The LCN2-positive cells were labeled with horseradish peroxidase developed with 3, 3′-diaminobenzidine (HRP-DAB), shown as the brown stain. The results were conducive to evaluating the mean intensity of LCN2-positive staining after BLM lung injury (Figure 1(e)). LCN2-positive cells were mainly found in the mouse alveolar epithelium and inflammatory cells. Interestingly, compared with the control group, the number and intensity of LCN2-positive cells were significantly increased after lung injury on Day 28, as indicated by black arrows in Figure 1(d). Meanwhile, a semiquantitative analysis of positively stained regions was conducted using the average optical density (AOD). The AOD of LCN2-positive regions in lung tissue post-BLM Day 28 was over 40% versus 26% on post-BLM Day 0 (Figure 1(e)).

### 3.2. The Type 2 Alveolar (AT2) Epithelial Cells in BLM-Injured Lungs Showed High LCN2 Expression

**Figure 2:**
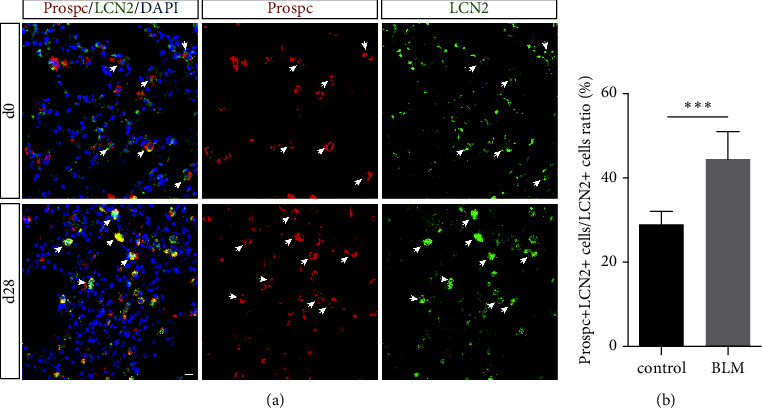
Highly expressed Lcn2 is mainly concentrated on alveolar type 2 cells in the BLM injury lung. (a) Mice lungs at Day 0 and Day 28 post-BLM injury were collected for immunofluorescent staining of Prospc and LCN2. The white arrow refers to LCN2^+^Prospc^+^ cells. (b) The ratio of LCN2^+^prospc^+^ cells to LCN2^+^ cells (%) was acquired by cell counting. Data are shown as mean ± SEM, *n* = 10; ^*∗∗∗*^*P* < 0.01 vs. the control group. Scale bar, 100 *μ*m.

LCN2 mRNA is expressed both in human lung tissue, including the trachea, and in the alveolar type 2 pneumocyte-derived cell line A549 [13]. Prospc has been proven to be an important biomarker of AT2 cells [14]. To determine the location of LCN2 in alveolar epithelial cells, we performed immunofluorescence staining of Prospc and LCN2. The green fluorescence signal indicated LCN2-positive cells, and the red fluorescence signal indicated Prospc-positive cells. Immunoblotting revealed that LCN2 and Prospc double-positive cells were LCN2^+^ AT2 cells, as indicated by white arrows. The number and intensity of LCN2^+^ cells increased in BLM-treated mouse lungs compared with those in control mice (Figure 2(a)). This observation was consistent with recent studies, revealing that LCN2 was upregulated in the epithelium in response to inflammation [15, 16]. We quantified the labeling efficiency of LCN2^+^AT2 cells by counting LCN2^+^ Prospc^+^ cells and Prospc^+^ cells, and the average labeling efficiency of LCN2^+^AT2 cells in mouse lung tissue post-BLM injury was 44.5% compared with 29.0% in control mice (Figure 2(b)).

### 3.3. LCN2 Impaired the Proliferation Capacity of Human Pulmonary Alveolar Epithelial Cells (HPAEpiCs) In Vitro

**Figure 3:**
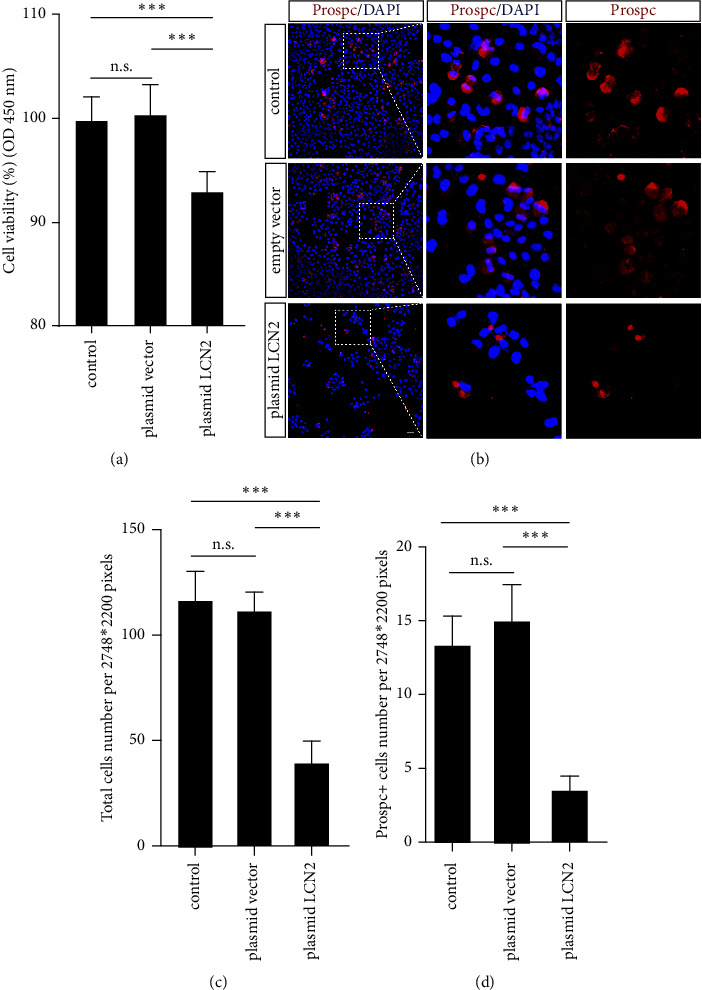
Overexpressed LCN2 impaired the proliferation capacity of human pulmonary alveolar epithelial cell (HPAEpiC) in vitro. (a) Effects of LCN2 on the proliferation ability of pulmonary alveolar epithelial cells (HPAEpiCs) were determined by CCK-8 assay. HPAEpiCs in the plasmid LCN2 group showed impaired proliferation ability compared to cells in the control group and the plasmid vector group (^*∗∗∗*^*p* < 0.01 vs. the control group and plasmid vector group; n.s., nonsignificant). (b) HPAEpiCs from the plasmid LCN2 group, control group, and plasmid vector group were collected for immunofluorescent staining of Prospc and LCN2. (c) Total cells per 2748 *∗* 2200 pixel were acquired by cell counting. (d) The Prospc^+^ cells per 2748 *∗* 2200 pixel were acquired by cell counting. The cell number in plasmid LCN2 declined significantly as compared to the control group and plasmid vector group (data are shown as mean ± SEM, *n* = 3; ^*∗∗∗*^*P* < 0.001 vs. the control group. Scale bar, 100 *μ*m).

To investigate the effect of LCN2 on the proliferation of alveolar epithelial cells, HPAEpiCs were selected and cultured in vitro. LCN2 was overexpressed in HPAEpiCs by plasmid transfection, and empty vector-transfected cells appeared to have negligible effects. The proliferation capacity of HPAEpiC in vitro was detected by CCK8 assay, and the cell viability was presented as optical density (OD450 nm) measurements using a microplate reader with a 450 nm filter. The cell viability of the plasmid LCN2 group decreased significantly compared with empty vector-transfected cells and negative control cells, whose OD measurements declined to nearly 90 (Figure 3(a)).

Then, immunofluorescence staining was performed to observe the cell growth, proliferation, and aggregation of all HPAEpiCs and Prospc^+^ HPAEpiCs. DAPI (4′, 6-diamidino-2-phenylindole) acted as a specific fluorochrome to stain the cell nucleus, and we realized that the plasmid LCN2 group had worse cellular growth behavior than the empty vector-transfected cells and negative control cells. The cell cluster of LCN2-overexpressing HPAEpiCs was obviously decreased, and the cell growth capacity was distinctly decreased (Figure 3(b)). The total HPAEpiC number per 2748 *∗* 2200 pixels was quantified, and the number of plasmid LCN2 group cells decreased significantly over half that of control cells (Figure 3(c)). AT2 cells have many metabolic properties, serving as cells with the potential to restore the alveolar epithelium after lung injury and prevent alveoli from collapsing upon exhalation [17]. To reveal the possibility of AT2 cells, Prospc staining was performed, and the Prospc^+^ HPAEpiC cell number was quantified. As expected, a number of Prospc^+^ HPAEpiCs were observed among all normal HPAEpiCs; nevertheless, Prospc^+^ cells were affected by LCN2 overexpression (Figure 3(b)). Meanwhile, the number of Prospc^+^ cells per 2748 *∗* 2200 pixels was counted for quantitative analysis, and the Prospc^+^ cell number decreased to less than 5 compared with control cells (Figure 3(d)).

### 3.4. IL-17 Reversed the Proliferation of HPAEpiCs Restrained by LCN2 and Showed a Partially Concentration-Dependent Effect

**Figure 4:**
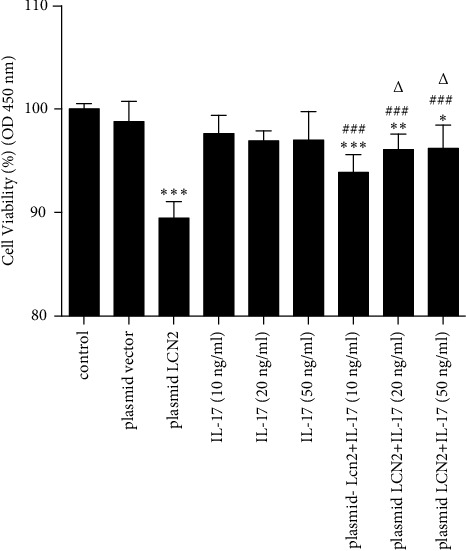
IL-17 mildly reversed the impaired proliferation of HPAEpiCs restrained by LCN2 in vitro and showed partial concentration dependence. The HPAEpiCs were intervened by rhIL-17 of different concentrations (10 ng/ml, 20 ng/ml, and 50 ng/ml) in vitro, and the effects of LCN2 on the proliferation ability of HPAEpiCs were determined by CCK-8 assay. IL-17 reversed the impaired proliferation ability of HPAEpiCs in plasmid LCN2 group but showed partial concentration dependence (data are shown as mean ± SEM, *n* = 3; ^*∗*^*P* < 0.05, ^*∗∗*^*P* < 0.01, ^*∗∗∗*^*P* < 0.001 vs. control group. ^###^*P* < 0.001 vs. plasmid LCN2 group. ^△^*P* < 0.05 vs. plasmid LCN2^+^IL-17 (10 ng/ml)).

To observe the effect of IL-17 on the proliferation of static HPAEpiCs and whether it has a concentration-dependent effect, different gradient concentrations of rhIL-17 (10 ng/ml, 20 ng/ml, and 50 ng/ml) were selected for the intervention experiment in vitro, and the cell viability of HPAEpiCs was examined by CCK8 assay. However, IL-17 showed little effect on the cell viability of HPAEpiCs compared with the control group and had no concentration-dependent effect. The OD values of HPAEpiC treated with IL-17 were close to those of the negative control and empty vector cells (Figure 4). The effect of different concentrations of IL-17 on cell viability was also used to determine whether the effect was concentration-dependent, and a slight effect on cell viability was found. Interestingly, as shown in Figure 4, IL-17 rescued the impaired viability of HPAEpiC cells induced by LCN2 overexpression. To a certain extent, different concentrations of IL-17 treatment for the plasmid LCN2 group were partially concentration-dependent; rhIL-17 at 20 ng/ml showed a more obvious intervention effect than rhIL-17 at 10 ng/ml, but rhIL-17 at 50 ng/ml did not show a significantly increasing effect (Figure 4). Therefore, we regarded rhIL-17 at 20 ng/ml as the better dose for further studies.

### 3.5. IL-17 in Moderate Dosage Stimulated the Proliferation and Cell Cluster of HPAEpiCs Inhibited by LCN2

**Figure 5:**
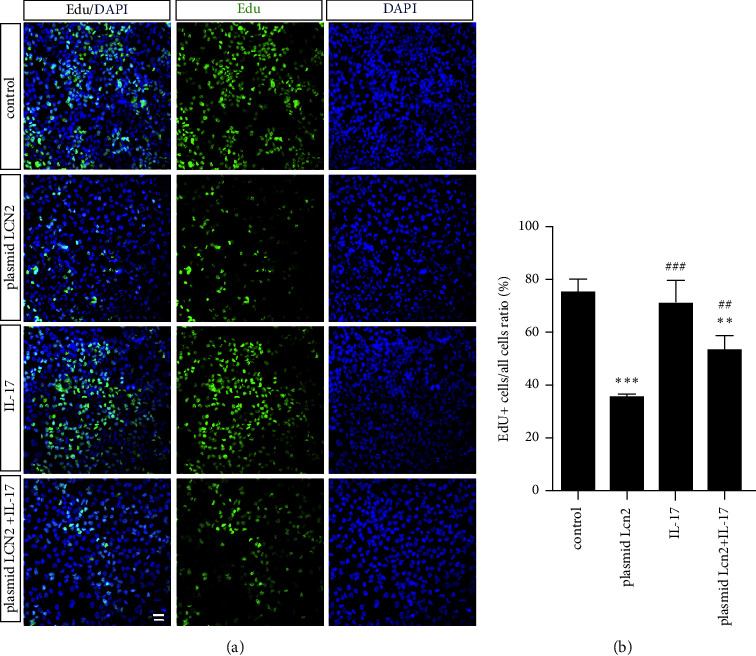
IL-17 in moderate dosage stimulated the proliferation and cell aggregation of HPAEpiCs inhibited by LCN2. (a) The proliferation of HPAEpiCs was measured by EdU (5-ethynyl-2′-deoxyuridine) assay at the cellular DNA level. The green fluorescence signal, acted as EdU-positive cells, presented proliferating cells. (b) The ratio of EdU^+^ cells to total cells (%) was acquired by cell counting (data are shown as mean ± SEM, *n* = 3; ^*∗∗*^*P* < 0.01, ^*∗∗∗*^*P* < 0.001 vs. control group. ^##^*P* < 0.005, ^###^*P* < 0.001 vs. plasmid LCN2 group. Scale bar, 100 *μ*m).

Next, cell proliferation can also be measured at the DNA level by an EdU (5-ethynyl-2′-deoxyuridine) assay. EdU covalently cross-linked to the fluorescent azide iFluor-488. Therefore, the green fluorescence signal, indicating EdU^+^ cells, indicated proliferating cells. The number of EdU^+^ cells in the plasmid LCN2 group significantly decreased compared with that in the control group (Figures 5(a) and 5(b)). Meanwhile, to observe the effect of IL-17 on the proliferation of HPAEpiCs, rhIL-17 at 20 ng/ml was used to intervene in cells. To some extent, the impaired proliferation capacity of the plasmid LCN2 group was partially rescued by IL-17 intervention compared with that of the plasmid LCN2 group. IL-17 intervention had no significant effect on the proliferation of HPAEpiC cells under normal conditions compared with control cells (Figures 5(a) and 5(b)).

### 3.6. IL-17 Mildly Rescued the Proliferative Capacity of Prospc-Positive Alveolar Epithelial Cells Impaired by LCN2 In Vitro

Given the above results that IL-17 rescued impaired cell proliferation induced by LCN2, we further predicted the type of alveolar epithelial cells affected in vitro. Immunofluorescence staining was performed to observe the cell growth of all HPAEpiCs and Prospc-positive HPAEpiCs.

**Figure 6:**
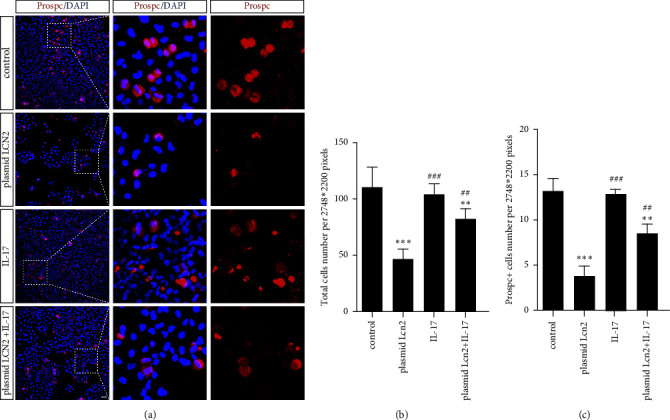
IL-17 rescued the proliferative capacity of Prospc-positive alveolar epithelial cells impaired by LCN2 in vitro. (a) HPAEpiCs from the control group, plasmid LCN2 group, IL-17 group, and plasmid LCN2^+^IL-17 group were collected for immunofluorescent staining of Prospc and LCN2. (b) Total cells per 2748 *∗* 2200 pixel were acquired by cell counting. (c) The Prospc-positive cells per 2748 *∗* 2200 pixel were acquired by cell counting. The cell number in the plasmid LCN2 group declined significantly as compared to the control group, and the cell number in the plasmid LCN2^+^IL-17 group increased as compared to the plasmid LCN2 group (data are shown as mean ± SEM, *n* = 3; ^*∗∗*^*P* < 0.01, ^*∗∗∗*^*P* < 0.001 vs. control group. ^##^*P* < 0.005, ^###^*P* < 0.001 vs. plasmid LCN2 group. Scale bar, 100 *μ*m).

After the rhIL17 treatment, the decreased proliferation capacity of the plasmid LCN2 HPAEpiC group was obviously rescued, and cell aggregation was more pronounced. Meanwhile, the number of Prospc^+^ HPAEpiCs in the plasmid LCN2 group treated with rhIL17 was obviously increased compared with that in the plasmid LCN2 group (Figure 6(a)). However, the rhIL-17-treated group did not show a significant decrease in cell proliferation compared with the control group (Figure 6(a)). Then, we quantitatively analyzed cell growth by counting the number of total HPAEpiCs and Prospc^+^ HPAEpiCs per 2748 *∗* 2200 pixels. The number of plasmid LCN2 group cells after rhIL17 treatment increased significantly compared with the plasmid LCN2 group (Figure 6(b)). Therefore, IL-17 partially reversed the proliferative capacity of Prospc^+^ alveolar epithelial cells impaired by LCN2 in vitro, which indicated that IL17 may play a role in alveolar epithelial repair after lung injury.

## 4. Discussion

In this study, we first established a BLM-induced lung injury in mice to observe the expression of LCN2 in alveolar epithelial cells. The results confirmed that LCN2 expression mainly focused on the alveolar epithelium and inflammatory cells of lung injury mice (Figures 1(d) and 1(e)), which was consistent with a previous study on LCN2 expression in chronic lung diseases, demonstrating that LCN2 was significantly upregulated in lung tissue after exposure to damage factors, such as ultrafine carbon particles, pathogens, and inflammatory cytokines [7, 8, 18]. Then, we further evaluated and quantified the expression of LCN2 in alveolar epithelial cells, and LCN2 and Prospc double-positive cells were highly expressed in lung tissue after BLM lung injury (Figures 2(a) and 2(b)). Given the regenerative capacity of AT2 cells, we hypothesized that LCN2 may affect the regenerative capacity of AT2 cells, thereby affecting the repair process of lung tissue. This hypothesis will be investigated by our future research, and it will be interesting to see the proliferative ability of alveolar epithelial cells.

LCN2 is involved in oncogenesis and progression in a variety of cancers and negatively modulated cell proliferation and epithelial-mesenchymal transition (EMT) by regulating energy metabolism-related gene expression in colorectal carcinoma [19]. In addition, glioblastoma multiforme (GBM) cells overexpressing LCN2 showed significantly decreased proliferation and invasion capacities [20]. These concepts were supported by our results on the cell viability of HPAEpiCs via CCK8 assay, indicating that LCN2 overexpression in HPAEpiC negatively impacted the cell viability of HPAEpiC (Figure 3(a)). Meanwhile, the cell growth state and Prospc expression of LCN2-overexpressing HPAEpiC were impaired and decreased (Figures 3(b), 3(c), and 3(d)). The above points indicated that LCN2 negatively regulates the proliferation of alveolar epithelial cells.

IL-17 is a proinflammatory factor secreted by Th17 cells that participates in promoting the proliferation of various cells in lung diseases, including fibroblasts in lung fibrosis [21], epithelial cells in lung cancer [22], and pulmonary arterial endothelial cells in pulmonary hypertension [23]. LCN2 serves as a downstream protein of the IL-17/NF-*κ*B pathway, which can be regulated by IL-17 [24]. The above concepts were verified by our investigation of the viability of HPAEpiC cells overexpressing LCN2 by plasmid transfection and treatment with different concentrations of rhIL-17. IL-17 had no obvious effect on the viability of HPAEpiCs but affected cells overexpressing LCN2, which promoted an increased trend of proliferation activity in a partially concentration-dependent manner when treated with different concentrations of rhIL-17 (Figure 4). In addition, immunocytochemical staining of HPAEpiCs was performed to detect the proliferative capacity of Prospc-positive cells (Figures 5(a) and 5(b)). IL-17 may partially reverse the decreased cell proliferation induced by LCN2 overexpression, and the cell type affected may be AT2 cells. Further studies are needed for in vivo confirmation.

Our findings above provide potential information about the effect of LCN2 on the decreased proliferation of the alveolar epithelium, especially that of AT2 cells, demonstrating possible clues that IL-17 partially reverses this phenomenon and revealing the potential mechanism by which LCN2 affects alveolar epithelial regeneration.

However, our previous findings have certain limitations. In our in vitro experiments, we utilized HPAEpiC cells which consist of both AT1 and AT2 cell types but failed to adequately elucidate the behavior of AT2 cells. Actually, these current studies are only considered to be suggestive observations of AT2 cells. In view of this limitation, our future study will involve a comprehensive analysis of the impact of LCN2 on the behavior of AT2 cells by isolating them from mouse and human lung tissue. This study provides direction and clues for further mechanistic research, highlighting an encouraging direction forward for exploring potential therapies for pulmonary fibrotic disease.

## Data Availability

The raw data supporting the conclusion of this article will be made available by the authors upon reasonable request.
